# Analysis and Comparison of New-Born Calf Standing and Lying Time Based on Deep Learning

**DOI:** 10.3390/ani14091324

**Published:** 2024-04-29

**Authors:** Wenju Zhang, Yaowu Wang, Leifeng Guo, Greg Falzon, Paul Kwan, Zhongming Jin, Yongfeng Li, Wensheng Wang

**Affiliations:** 1 Agricultural Information Institute, Chinese Academy of Agricultural Sciences, Beijing 100086, China; zhangwenju@caas.cn (W.Z.); guoleifeng@caas.cn (L.G.); jinzhongming@caas.cn (Z.J.); liyongfeng_1116@163.com (Y.L.); 2 Laboratory of Geo-Information Science and Remote Sensing, Wageningen University & Research, 6708 PB Wageningen, The Netherlands; 3 College of Science and Engineering, Flinders University, Adelaide, SA 5042, Australia; greg.falzon@flinders.edu.au; 4 School of Science and Technology, University of New England, Armidale, NSW 2351, Australia; 5 School of Engineering and Technology, College of ICT, Central Queensland University, Rockhampton, QLD 4701, Australia; w.kwan@cqu.edu.au

**Keywords:** behaviour monitoring, animal welfare, deep learning, health indicator

## Abstract

**Simple Summary:**

In the process of calf rearing, it is inevitable to encounter issues of illness and death among calves. Often, due to the inability to detect sicknesses such as diarrhoea in a timely fashion, these sicknesses lead to the calves’ demise. This research starts from the practical application needs, and proposes the development of a monitoring system using deep learning technology to monitor the daily standing and lying behaviour of calves to predict their condition and adaptation to the environment. By analysing the standing and lying time of calves, the system can provide early warnings about calves’ condition and health status. This research helps to promptly grasp calves’ condition and growth status, thereby improving their welfare and management, enhancing the health condition of reared calves, ensuring the quality and safety of meat and milk, and reducing production costs. This research method also offers a new idea for the construction of smart ranches, as the construction of precision and smart ranches is not only a demand of consumers but also an inevitable direction for the development of the breeding industry.

**Abstract:**

Standing and lying are the fundamental behaviours of quadrupedal animals, and the ratio of their durations is a significant indicator of calf health. In this study, we proposed a computer vision method for non-invasively monitoring of calves’ behaviours. Cameras were deployed at four viewpoints to monitor six calves on six consecutive days. YOLOv8n was trained to detect standing and lying calves. Daily behavioural budget was then summarised and analysed based on automatic inference on untrained data. The results show a mean average precision of 0.995 and an average inference speed of 333 frames per second. The maximum error in the estimated daily standing and lying time for a total of 8 calf-days is less than 14 min. Calves with diarrhoea had about 2 h more daily lying time (*p* < 0.002), 2.65 more daily lying bouts (*p* < 0.049), and 4.3 min less daily lying bout duration (*p* = 0.5) compared to healthy calves. The proposed method can help in understanding calves’ health status based on automatically measured standing and lying time, thereby improving their welfare and management on the farm.

## 1. Introduction

Along with an increasing quality of life, concerns about milk quality have increased the interest in cows’ physical and emotional welfare during breeding [1]. A potential approach to assess cows’ welfare status is to monitor their behaviours. Apart from observing their abnormal behaviours such as fighting and aggression, monitoring their normal animal behaviours like eating, standing, walking, and lying is becoming popular, and could be used to analyse the physical condition of cows and the impact of the external environment on them, therefore monitoring their health, emotions, and well-being [2].

Monitoring cows’ behaviour, including standing and lying, has undergone several phases. It began with traditional manual observation and then progressed to the use of electronic accessories. For example, Chapinal et al. used pedometers to monitor standing and lying behaviours [3]; Jaeger et al. used electronic ear tags to monitor cow lying behaviour [4]; and Khanh et al. used 3-axis acceleration to classify lying, standing, and feeding behaviours [5]. However, these accessories, which must be attached to cows, are invasive and could not only be missed or damaged but could also cause injury and stress to the cows [6].

Recently, cows’ standing and lying behaviour was non-invasively monitored through computer vision. Researchers have used deep learning techniques to detect individual cows’ standing and lying behaviour. The behaviour recognition of cows by computer vision technology is not only conducive to the good management of cows on dairy farms, but also more conducive to the welfare of cows due to its non-invasive characteristics. Fuentes et al. used deep learning networks to detect the standing and lying behaviour of cows and compared the precision of different algorithms [7]. Computer vision algorithms can also be used to monitor the respiratory behaviour of multiple cows [8,9], as well as to identify and track the movements of dairy cows [10]. However, studies are relatively insufficient for new-born calves, whose growth significantly affects subsequent milk production quality. To monitor calf health conditions, standing and lying behaviour are important indicators [11,12,13]. Goharshahi et al. used accelerometer ear tags to monitor diarrhoea in female calves and found that calves with diarrhoea spent an average of 64.8 min longer lying down than healthy calves on the day before clinical identification [14]. Until now, few studies have researched automated monitoring and statistical analysis of calf behaviour’s duration using deep learning technology. Therefore, the research problem is to explore a method that uses deep learning technology to monitor the time calves spend standing and lying down as a predictor of calf health, thereby enabling real-time monitoring of calves to improve the level of calf-rearing management.

The aim of this study was threefold: (1) to establish an identification system based on deep learning algorithms to recognise both standing and lying behaviours of new-born calves, (2) to predict the standing and lying time of new-born calves on a daily basis, and (3) to compare the daily performance of healthy calves to those calves afflicted with diarrhoea. The results of this study would assist in the development of a computer vision system which could help breeders to promptly identify new-born calves with abnormal standing and lying behaviours compared to healthy calves.

## 2. Materials and Methods

All protocols involving animals were approved by the Experimental Animal Care and Use Committee of the Institute of Animal Sciences, Chinese Academy of Agricultural Sciences, Beijing, China (approval number IAS2021-220).

**Figure 1:**
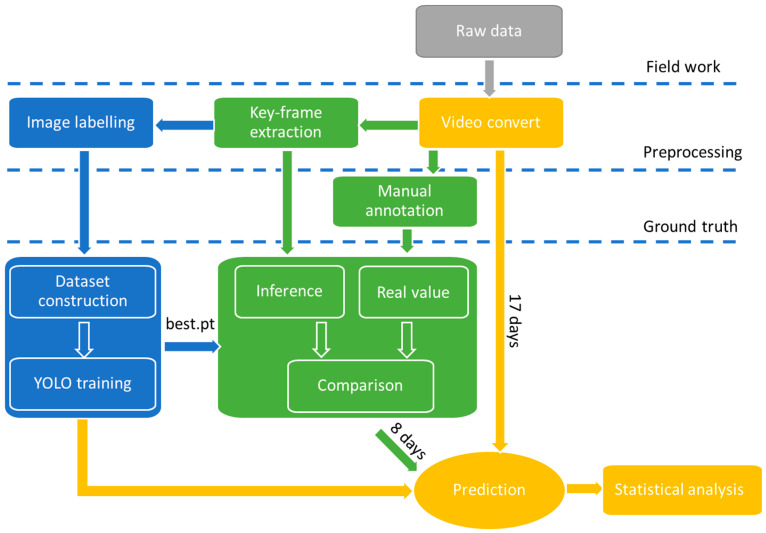
The flowchart of the research.

This section explains the process of data acquisition, the data preprocessing steps, the dataset construction approach, and the implementation of the calf-behaviour prediction method. Figure 1 shows the flowchart of the entire procedure.

### 2.1. The Setup of Experiments

**Figure 2:**
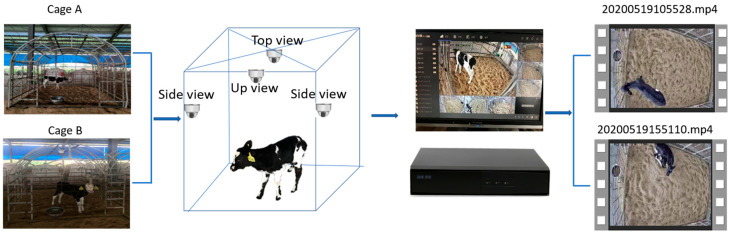
Schematic of the video recording process.

Fieldwork was conducted from 19 to 24 May 2021, at Yinxiangweiye Dairy Farm in Shandong Province, China, where more than 1000 Holstein cows and around 100 calves were raised. New-born (born within 15 days) calves were separately housed in calf cages, where they were disturbed at the lowest limit. For this study, six new-born calves were randomly selected, including four healthy calves (ID: 9350, 9347, 9348, 9349) and two sick calves (ID: 9389, 9351) (Table 1). All new-born calves participating in the experiment were reared under the same conditions as other new-born calves on the farm during the experimental period. Calves that were not found to have any disease throughout the experiment were defined as healthy calves. Calves with diarrhoea were classified as sick calves. Diarrhoea was diagnosed based on faeces observation and an elevated rectal temperature. Two types of cages are used on the farm, which are cage A and cage B. Cage A is 3 m long, 2.5 m wide, and 3 m high. Cage B is 2.5 m long, 2 m wide, and 2 m high. The floors of both were bedded with a 15 cm thick layer of dry rice bran that was refreshed every 25 days. During the six recording days, five calves were housed in cage type A, while the other calf was housed in cage type B (Figure 2). They were all provided with unlimited forage and fed 4-L-milks with a bucket twice a day at 7 a.m. and 5 p.m., respectively, and given 300 millilitres of water with a bucket at 7 a.m., 12 a.m., and 5 p.m., respectively. There was a ventilation system in the shed. The daily average temperature and humidity index ranged from 73.14 to 81.06 during the experiment. Our experiment did not change the daily living environment of the calf, but only added a camera outside the calf cage to collect data.

Figure 2 illustrates the procedure of data acquisition. Raw data were acquired by using six Hikvision cameras (DS-3347WD-L, Hikvision Digital Technology Co., Ltd., Hangzhou, China) with a resolution of 2580 × 1440 pixels at 25 HZ. Because this study requires 24/7 data collection, it is necessary to ensure that the quality of the data collected during the day and at night is the same. Therefore, it is necessary to choose a camera with an infrared camera function to collect data. Moreover, due to lighting reasons, to obtain high-quality nighttime data it is necessary to choose a higher-resolution camera. This camera has an infrared camera function and high resolution, and the price is relatively low. To consider the field of view over the full cage, each camera was deployed at one of four spots in the cages to record data (details are given in Experimental setup instructions (Supplementary Materials)). The raw data were 1080 videos, each with a length of approximately 49 min, resulting in 30 videos representing a recording of 1 calf-day.

### 2.2. Dataset Construction

A dataset of 3406 images was constructed. Images were selected following four conditions: (1) each calf in the experiment was covered, (2) all four recording angles were included, (3) abundant patterns of lying and standing behaviours were involved, (4) and scenes under various illumination strengths were recorded. To build the dataset, 330 videos were manually selected. From each video, key-frames that acted for the action transitions of calves were extracted by OpenCV using a diff function. Then, a manual selection was carried out to reduce duplication. The dataset comprises 1700 images of standing and 1706 images of lying. Images were labelled as “standing” or “lying” according to their content by using LabelImg (version: 1.8.6). Labels were saved in the format of txt format that was suitable for the training of YOLO. Finally, the dataset was split into training and validation sets in a ratio of 9.5 to 0.5 by shuffling and selecting images.

### 2.3. Model Training

**Figure 3:**
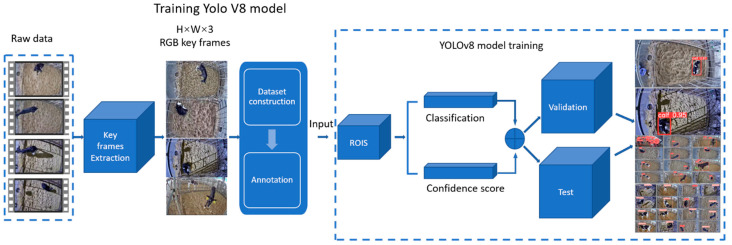
Flowchart of the model training process.

To classify the standing and lying behaviour of calves, a YOLOv8n model was trained via transfer learning. Compared to the preceding YOLO versions, YOLOv8 has structural and architectural improvements and can recognise dynamic objects faster and more accurately. Among the YOLOv8 series, YOLOv8n, as the smallest model, whose size is less than 10 MB, runs the fastest, which is very consistent with our need to observe the standing and lying behaviour of calves in real time. The batch size and epochs were initialised as 48 and 300, respectively. The model was trained on a computer with an 8-core CPU and a 16GB Tesla T4 GPU. Figure 3 demonstrates the training pipeline.

#### 2.3.1. Loss Function

The loss values of YOLOv8n in the recognition of standing and lying behaviour of calves include the classification loss of standing and lying behaviour ( ${Error}_{cls}$ ), distribution focal loss ( ${Error}_{dfl}$ ), and bounding box location loss ( ${Error}_{bbox}$ ), respectively. ${Error}_{cls}$ is the loss calculated from the difference between the predicted probabilities and the actual class labels. It measures how well the model is able to classify each object. ${Error}_{dfl}$ is a modified version of focal loss used to address class imbalance by focusing more on hard-to-classify examples. It adjusts the focus based on the correctness of the class probability distribution, helping to prioritise the learning on misclassified instances. ${Error}_{bbox}$ measures the difference between the predicted bounding boxes (which define the location and size of the object) and the actual ground truth bounding boxes. It is crucial for accurately determining where objects are located in the image. The loss function is shown in Formula (1).

(1) $$\begin{array}{c} Loss={Error}_{cls}+{Error}_{dfl}+{Error}_{bbox} \end{array}$$

IOU is the intersection over the union of A (the real bounding box of the frame) to B (the predicted bounding box of the frame). The symbols “∩” and “∪” represent mathematical operations on A and B : “∩” denotes the intersection of A and B . In the case of bounding boxes, it refers to the area that is overlapped with both the predicted bounding box ( B ) and the actual bounding box ( A ). “∪” denotes the union of A and B . For bounding boxes, it refers to the total area covered by both bounding boxes combined by either bounding box ( A ) or bounding box ( B ), which is defined as Formula (2)‚

(2) $$IOU=\frac{A\cap B}{A\cup B}$$

#### 2.3.2. Evaluation Metrics

This work uses F1 -score and mean Average Precision ( mAP ) as the evaluation metrics to evaluate the model performance of YOLOv8n for calf standing and lying behaviour classification and recognition. The evaluation metric formulas are shown in the following equations,

(3) $$Precision=\frac{TP}{TP+FP}$$

(4) $$Recall=\frac{TP}{TP+FN}$$

(5) $$F1=\frac{2}{{Precision}^{-1}+{Recall}^{-1}}$$

where TP denotes the number of True Positives, FP denotes the False Positives, and FN denotes the False Negatives.

Average precision ( AP ) is one of the standard metrics to evaluate the sensitivity of the model recognition, which is calculated using the Precision–Recall curve (*PR*), which describes both precision and recall for different object detector confidence thresholds. The AP metric is calculated as Formula (6).

The mean average precision ( mAP ) is the average of AP values for all classes; the formula is shown in (7). In this application there are classes. The higher the mAP value, the better the object detector performance. Different mAP metrics can be specified based on the level of detecting bounding box overlap with the ground truth: mAP50 denotes the average of AP when the IOU ≥ 50%, and mAP50–95 denotes 50% ≤ IOU ≤ 95%.

(6) $$AP=\int_{0}^{1}P_{\left(R\right)}dR$$

(7) $$mAP=\frac{\sum_{i=1}^{C}{AP}_{i}}{C}$$

Frames per second ( FPS ) denotes the prediction speed of the model, N is the total number of the predicted video frames, and $t_{N}$ is the total time taken by the model to predict the video. The FPS formula is shown in (8).

(8) $$FPS=\frac{N}{t_{N}}$$

### 2.4. External Validation on 8 Calf-Days of 24 h Labelled Data

A trained model requires simple internal tests using a test set to verify its ability to classify. However, the performance of the test set does not guarantee the applicability of our system in practice and the generalisability of the model cannot be demonstrated. To prove that the model can be used in practical application scenarios, it is very important to verify the reliability and the effectiveness of the proposed method in practice [15]. Therefore, the performance of the model on the test set is not described in this paper. To verify the reliability of the YOLOv8n-based system in practice, the study randomly selected 8 calf-days of 24 h video data collected in a real breeding environment to manually count the daily standing and lying time of calves (for ground truth see Table 2). Then, 4 out of 8 calf-days’ data with the maximum predictive error were selected, manually annotated with daily standing and lying behaviour, and visualised using scatter plots. The scatter plots of these 4 calf-days were compared with the predicted results of the system to demonstrate the reliability of the system. Table 2 lists the details of the ground-truth annotation.

Ground truth represents the total time of 30 videos within 24 h (camera display time) from the start of video recording. The reason why the total time here is not equal to 24 h is that the camera automatically saves a day’s videos into about 49 min each, in total 30 videos a day. However, when saving between videos, some videos overlap and some videos lose frames, resulting in a duration not exactly equal to 24 h.

Due to the method’s use of the YOLOv8n classification to identify the standing and lying behaviour frames of calves, the formula for calculating the standing and lying time of calves in the system is defined as follows:

(9) $$Time=\sum_{i=1}^{30}\frac{{Frames\;of\;Video}_{i}}{{FPS}_{i}}$$

(10) $$Standing\;Time=\frac{Total\;number\;of\;Standing\;Frames}{FPS}$$

(11) $$Lying\;Time=\frac{Total\;number\;of\;Lying\;Frames}{FPS}$$

where Frames of Video_i_ represents the total standing and lying time of each calf in 30 videos within 24 h. FPS indicates the frame rate of the video, which is 25.

**Figure 4:**
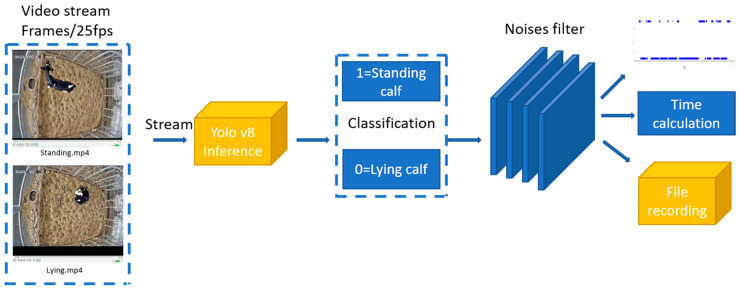
The system’s flow chart for predicting calf standing and lying time in the real environment.

This study used the YOLOv8n-based system to predict 8 calf-days’ daily standing and lying behaviour time video data of calves with the same data of the manually counted as mentioned above in order to calculate the corresponding time. As a demonstration of the system’s detection, this study also selected the behaviour distribution corresponding to manual annotations for 4 calf-days and output it as a scatter plot. These 4 calf-days’ distributions are used to compare with the scatter plot distribution of the corresponding ground truth. The system prediction flow chart is shown as Figure 4.

### 2.5. Automatic Prediction on 17 Calf-Days of 24 h Data

After verifying the generalisation ability of the system based on YOLOv8n, an automatic prediction of calves’ standing and lying behaviours was implemented to compare the differences in standing and lying time between healthy and sick calves. The study used this system to automatically predict six calves’ standing and lying time data for a total of 17 calf-days.

### 2.6. Statistical Analysis

Linear regression analysis was performed to verify the statistical relationship of the system prediction with the ground truth on the 8 calf-days of 24 h data. The results were shown with R^2^ and RMSE. When the effectiveness of the system was verified, the model was further used to predict untrained 17 calf-days’ data to explore the behavioural difference between healthy and sick calves. Statistical significance was defined at *p* < 0.05.

## 3. Results

In this study, we presented a calf standing and lying behaviour identification system based on the YOLOv8n algorithm that can quantitatively predict the daily standing and lying time of calves. The system was used to predict the daily standing and lying time of new-born calves for 17 calf-days. The daily standing and lying times of healthy and sick calves predicted by the system were then analysed and compared.

### 3.1. Model Performance

**Figure 5:**
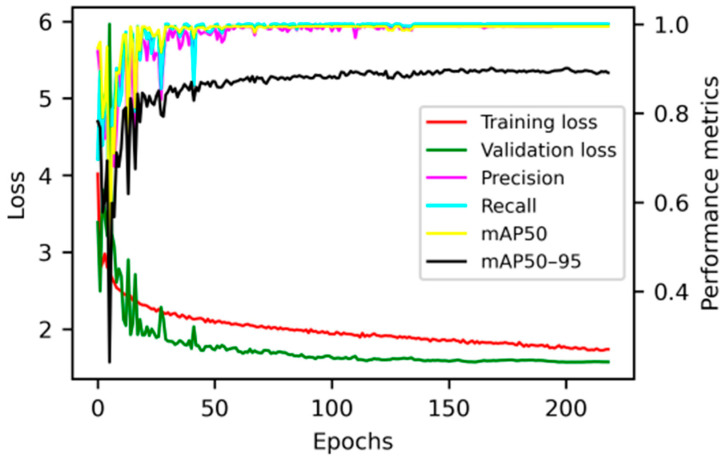
Training curves, training loss, and validation performance. Epochs refers to the number of times the entire dataset is passed through the neural network during training. Multiple epochs are often required to adequately train the model and optimise its performance on the dataset.

When the epochs reached 219, the model stopped training early as no improvement was observed. The training results are basically stable and the network converges so the training ends automatically, the precision obtained is excellent, and the loss value is minimised in the validation set. Figure 5 shows the performance of training loss, validation loss, precision, recall, mAP50 and mAP50–95 during the training and validation process, with the best model saved as best.pt.

**Figure 6:**
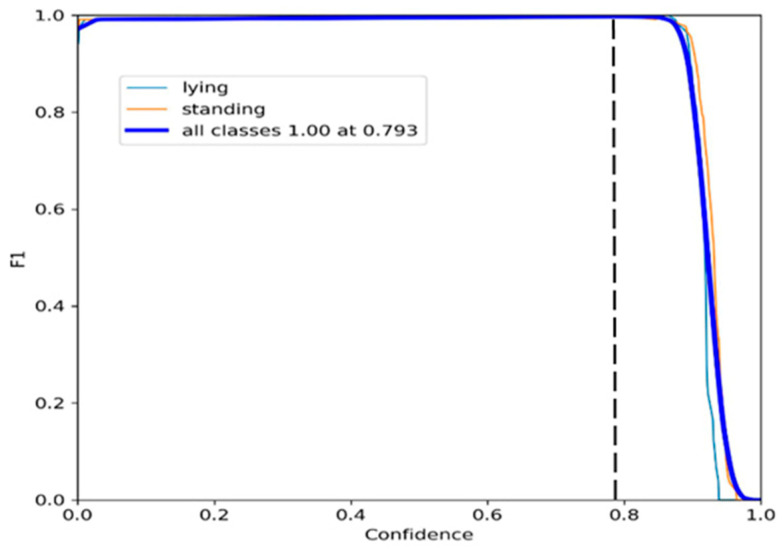
The performance of F1-confidence curve.

As shown in Figure 6, when the model achieved optimal precision in recognising calf standing and lying behaviours, the maximum F1 score was obtained at a confidence threshold of 0.793.

The best model was observed at epoch 169, at which the recall reached 1 and mAP50 reached 99.5%. Table 3 shows the performance of the validation set under the best model. In the 170 images of validation sets, there are 112 images of standing calves and 56 images of lying calves. Among these 170 validation images, two of them were occluded images that did not capture calves (in one image, the calf was severely obstructed by the breeder; in the other image, there was no calf, showing grey shadows). The validation results are all correct. Meanwhile, the recognition speed of the model can reach 333 FPS in the process of video recognition.

**Figure 7:**
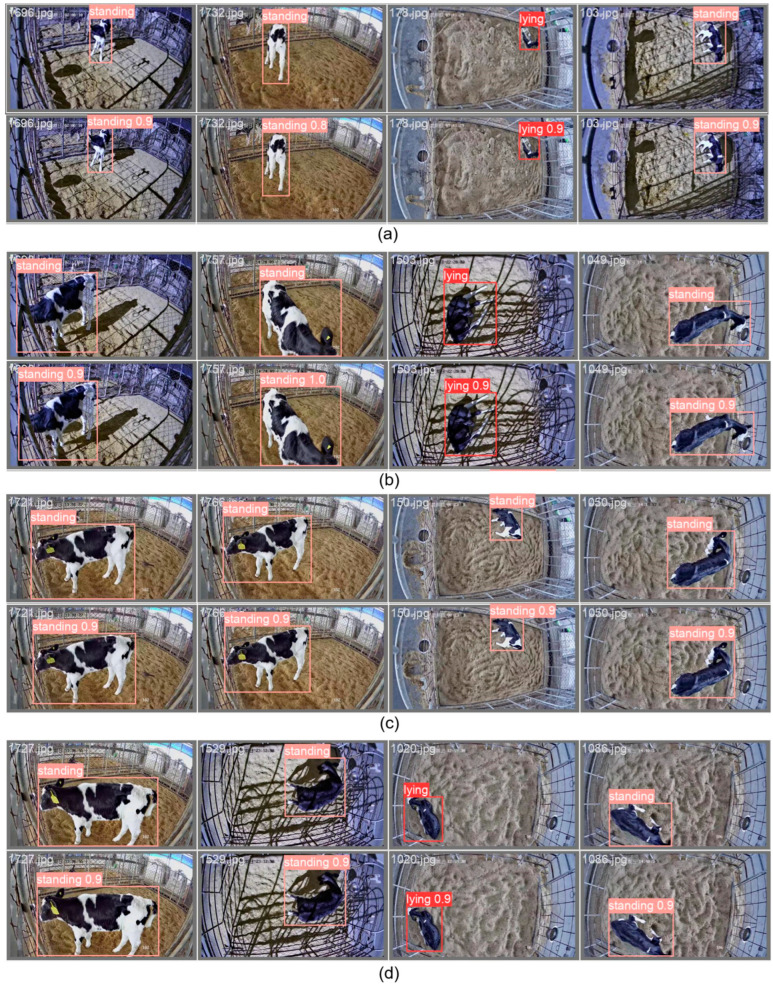
Four sets of images (**a**–**d**) represent the Qualitative results of 16 verified example frames. The manual annotations are given at the top of each set of images, while the predictions are at the bottom of each set.3.2. External Validation on 8 Days of 24 h Labelled Data.

Observing the classification results of the validation set data using the best model obtained, all images in the validation set showed correct recognition and classification of calves’ standing and lying behaviours. A random selection of 16 frames in the validation set is displayed in Figure 7 to demonstrate the prediction results.

### 3.2. External Validation on 8 Days of 24 h Labelled Data

#### 3.2.1. Prediction of Calf Standing and Lying Time over Untrained 8 Calf-Days

The largest error in daily standing time in the predicted results comes from Calf ID: 9351.19.12-20.12. The daily standing time is 592 s longer than the actual time, and the daily lying time is 799 s shorter than the actual time. The second largest error comes from the calf ID: 9350.23.11-24.11, where the daily standing time is 612 s less than the actual time and the daily lying time is 494 s more than the actual time. The third major error is ID: 9349.19.12-20.12. The daily standing time is 159 s shorter than the actual time, and the daily lying time is 79 s shorter than actual time. The error in daily standing and lying time for the other calf-days is within 211 s.

**Figure 8:**
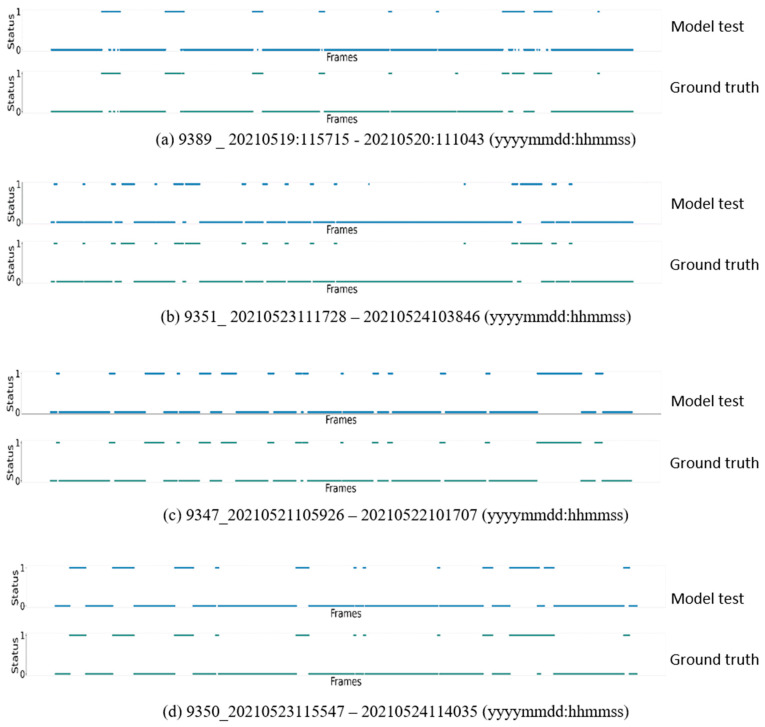
The 24 h behaviour distribution of the system’s automatic prediction and manually observed statistics in the four worst-predicted data segments (**a**–**d**). Standing and lying are represented by 1 and 0, respectively.

From the daily standing and lying behaviour time of six calves, with results predicted by the system for a total of 8 calf-days, it can be observed that the predicted values of the system are very close to the ground truth. To confirm whether the distribution of daily standing and lying behaviours predicted by the system is consistent with the ground truth, a scatter plot is illustrated in Figure 8 based on the 4 calf-days with the maximum predictive errors.

According to Figure 8, the ground truth and the prediction are largely consistent in behavioural distribution without significant misclassification between standing and lying behaviours, confirming the good predictive performance of the proposed method.

#### 3.2.2. OLS Regression Analysis on the Standing and Lying Time of Calves Based on the Ground Truth and Prediction over 8 Calf-Days of Data

**Figure 9:**
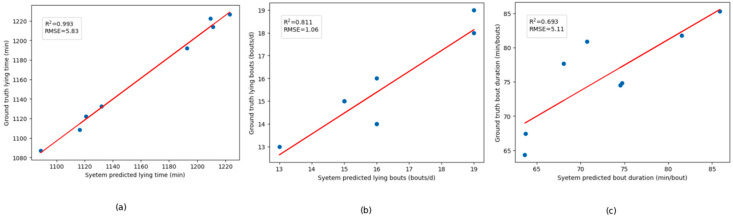
OLS regression analysis results of predicted lying time, lying bouts, and bout duration over 8-calf-days (**a**–**c**).

In the study, the Ordinary Least Squares (OLS) regression method was used to perform a regression analysis on the predicted and ground truth of the daily lying time, number of lying bouts, and average bout duration data of calves for different 8 calf-days. In the graph of each OLS regression analysis result, the horizontal axis represents the system predicted results, and the vertical axis represents the ground truth. The results showed that the OLS regression model fitted well. For the lying time, R^2^ is 0.993 and the Root Mean Square Error (RMSE) is 5.83; for the lying bouts, R^2^ is 0.811 and the RMSE is 1.06; for the bout duration, R^2^ is 0.693 and the RMSE is 5.11, which indicates that the predicted values of the system regarding the lying time of calves are very close to the ground truth, and the system has good predictive ability. The data validation of the standing and lying time of randomly selected calves in the total of 8 calf-days shows that the system is reliable. The OLS regression analysis results are shown in Figure 9.

### 3.3. Practical Implementation Using 17Calf-Days of 2 h Data

Due to the excellent performance that the proposed system demonstrated in the 8-calf-day application, it was further applied to predict the standing and lying behaviour time of calves collected in actual scenarios using 17 calf-days of video data on which the behaviour classification model was not previously trained, analysing the difference between the standing and lying time of sick calves and healthy calves based on the predicted results. The results showed that compared to healthy calves, calves with diarrhoea had about two hours less standing time per day, corresponding to about two hours more lying time per day, 2.65 more lying bouts per day, and 4.3 min less lying time bout duration.

**Figure 10:**
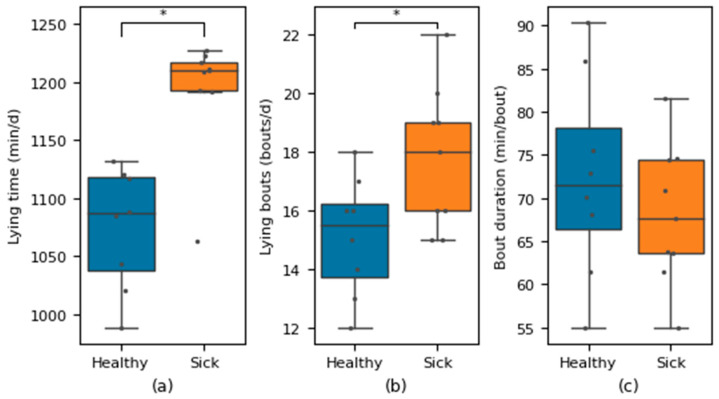
Daily lying time, number of lying bouts, and average bout duration using 17 calf-days of data. Differences between healthy and sick calves are denoted by * (*p* ≤ 0.05) (**a**–**c**).

When the Wilcoxon rank sum test was performed on the daily lying time, lying bouts, and bout duration data of calves, three *p*-values of 0.002, 0.049, 0.50 were obtained, respectively. The *p*-values of daily lying time and lying bouts are both less than 0.05, indicating a significant level of outcome. The bout duration *p*-value is more than 0.05, indicating that the results are not significant. This indicates that there are significant differences in daily lying time and number of lying bouts between healthy calves and calves with diarrhoea, and the difference in average bout duration is not significant between healthy calves and calves with diarrhoea. The results are shown in Figure 10.

## 4. Discussion

In this study, the aim was to achieve a robust behavioural classification and monitoring system for individually housed calves. The study used this system to predict the daily standing and lying time of new-born calves. It compared the daily standing and lying behaviour of healthy calves and calves with diarrhoea through predicted results. To achieve these aims, the ability of the trained deep learning model was validated through detecting daily standing and lying behaviour in real settings, including both healthy and sick calves. To the best of our knowledge, this study represents the first application of vision-based deep learning methods specifically to non-invasive monitoring of daily standing and lying behaviour time in calves.

### 4.1. Performance in Classifying Daily Standing and Lying Time of Calves

Generally speaking, the more training samples there are and the more diverse they are, the better the generalisability of the trained network will be. Therefore, we fixed the video cameras at different angles and heights to obtain diverse data for training YOLOv8 models. The results in the testing data show extremely good outcomes with almost no misclassification. The mAP was as high as 0.995, which is consistent with previous studies using YOLO networks to detect and classify cow behaviours [9,16]. Our work once again demonstrates the strong ability of YOLO algorithms in detecting animal behaviours.

As pointed out by Cheng et al. [15], there is a lack of practical applications in relevant studies. In this study, we validated the trained YOLO model by comparing the inference results with the ground truth on the daily standing and lying time using 8 calf-days’ data. The strong statistical relationship between the inference results and the ground truth suggests that our proposed method works effectively in classifying standing and lying behaviours on a daily basis.

When analysing the reasons for the misclassification, the 2 days’ data with the highest errors were all subject to blind spots during shooting. For example, for the side-view cameras whose field of view covered the length of the nearest side of the cages, the calf’s head protruding from the cage led to an incomplete calf body in frames and subsequent error in behavioural recognition. It should be noted that when installing cameras in the experiment, care must be taken to avoid blind spots in the field of view. The error in daily standing and lying time can be further lowered to less than 5 min when only summarising the 6 days’ data without blind spots. Thus, it can be speculated that the proposed method should work better with proper camera mounting in practice.

### 4.2. Behavioural Differences between Healthy and Sick Calves

As the proposed system had showed its performance in the 8-calf-days application, it was further applied to the entire 17 calf-days of data to gain insights into the classification of health conditions. New-born calves have been reported to have a decreased lying time with age [14]. Our healthy calves, aged 10 ± 2 days, had a median daily lying time of 17.9 h, which was consistent with the results of Goharshahi et al. [14] where calves of similar ages were used.

As expected, the healthy calves spent a shorter time lying than the sick calves by an average of 2 h per day. This result falls in line with, but is twice the difference of the study of Goharshahi et al., where calves with diarrhoea had a 67.2 min longer average daily lying time on the onset day compared with control calves [14]. In addition, the observed increased lying bouts in calves with diarrhoea are consistent with the study of Swartz et al. [17]. Indeed, an increased posture change is generally known as an indicator of restlessness and discomfort and can be witnessed more often in calves experiencing abdominal pain. It should be noted that some studies reported very different results in calves with diarrhoea, including decreased lying time and bouts, and decreased lying time but increased bouts [17,18,19].

This inconsistency among studies can be explained by the different pathogenesis, the severity of the outbreak, and the time of diagnosis [20]. For example, when systemic symptoms appear (e.g., fever, depression, decreased appetite), calves’ lying time increases significantly and they become lethargic [21,22]. In the present study, diarrhoea was diagnosed based on faeces observation and an elevated rectal temperature. Thus, the calves in our study with diarrhoea may have experienced a more severe infection. Additionally, our new-born calves could be more susceptible to severe disease responses compared with older calves used in previous studies. In summary, the key to an effective monitoring system for diarrhoea is to focus on the calf that is lying for an abnormal period of time, as both an increase and decrease can indicate the onset of diarrhoea.

### 4.3. Limitations and Future Work

In this study, the observation time of calves was short, and the sample size was limited, making it inadequate for time-series analysis of the disease process (non-occurrence, occurrence, progression). Our proposed vision-based, non-invasive approach has demonstrated its effectiveness for practical applications in monitoring lying and standing behaviours, without placing any burden on the calves. In the next step, we plan to apply it to conduct larger-scale and longer-term observations and develop a predictive model for calf diarrhoea based on the analysis of these observations. Farms only need to install the appropriate cameras to monitor the standing and lying behaviour of calves, thereby improving calf rearing. However, due to the generalisation issues of deep learning models, this method cannot be directly applied to different farm animals. When directly transferred to different farm settings, the accuracy decreases, necessitating incremental training with data from new scenes.

In addition, faeces samples were not collected in this study to confirm the pathogenesis of the diseases. Further investigation into the association between specific pathogens and calf behaviour will contribute to the development of a more comprehensive health monitoring system for calves.

## 5. Conclusions

In this study, we used six cameras from four angles to continuously record the daily standing and lying behaviour of six calves born within 15 days on a dairy farm. We trained a model based on the YOLOv8n CNN model to classify these behaviours. The feasibility of our model was tested by comparing the prediction with the ground-truth data of 8 calf-days, where the maximum difference is less than 14 min. Further, the model was used to predict the daily standing and lying time data of six calves collected in a real environment for a total of 17 calf-days. The predicted results demonstrated that healthy calves had about 2 h more daily standing time and 2 h less lying time than sick calves with diarrhoea. This indicates that diarrhoea symptoms can alter the daily standing and lying time of calves. This method demonstrates that using deep learning algorithms to establish models can be used for monitoring the standing and lying behaviour of new-born calves. Computer vision technology is gradually replacing traditional calf monitoring methods due to its non-invasive monitoring characteristics. The methods discussed can assist farm managers in monitoring calves. When abnormalities in standing and lying behaviours occur, the system can promptly detect them and allow for focused attention. Systematic monitoring can reduce calf mortality and production costs, as well as enhance calf health and welfare. Additionally, it provides technical support for the health management of dairy cows, optimisation of breeding, and the development of smart livestock farming.

Although our method has achieved good results, there are still some areas for improvement. This study only collected data from six calves for six days, which is insufficient to explain the changes in calves’ daily standing and lying time, and cannot establish standards for calves’ daily standing and lying time. Future work will focus on collecting more continuous data on calves to establish a standard for daily standing and lying time for healthy calves, which can be used for early warning of calf abnormalities.

## Figures and Tables

**Table 1:** The profiles of the six calves for data acquisition.

ID	Birth Date (m.dd)	Physical Condition	Weight (kg)	Cage Type
9389	5.15	Diarrhoea	30	A
9350	5.09	Healthy	38	A
9349	5.08	Healthy	36	A
9348	5.07	Healthy	36	A
9351	5.09	Diarrhoea	31	A
9347	5.07	Healthy	36	B

**Table 2:** Ground-truth standing and lying time of 8-calf-days’ data.

ID	Selected Date (dd.hh–dd.hh)	Start Time (yyyymmdd:hhmmss)	Standing Time (h:mm:ss)	Lying Time (hh:mm:ss)	Total Time (hh:mm:ss)
9347	21.10–22.10	20210521:105926	5:52:43	18:06:54	23:59:37
9350	23.11–24.11	20210523:115547	5:33:39	18:28:22	24:02:01
9348	19.12–20.12	20210519:123442	5:07:25	18:52:19	23:59:44
9349	19.12–20.12	20210519:121701	5:21:51	18:42:10	24:04:01
9389	19.11–20.11	20210519:115715	3:37:16	20:26:36	24:03:52
9389	21.16–22.16	20210521:162747	4:08:04	19:51:56	23:59:45
9351	19.12–20.12	20210519:122813	3:39:29	20:22:35	24:02:04
9351	23.11–24.11	20210523:111728	3:47:54	20:14:05	24:01:59

**Table 3:** The performance of the best model.

Model	Average Precision		mAP50	mAP50–95	FPS	Size (MB)
	Lying	Standing				
YOLOv8n	0.998	0.991	0.995	0.901	333	5.92

## Data Availability

The data presented in this study are available upon request from the corresponding author.

## Supplementary Materials

The following supporting information can be downloaded at: https://www.mdpi.com/article/10.3390/ani14091324/s1, Figure S1. Two types of cages used on the farm. (a) type A is 3 m long, 2.5 m wide, and 3 m high. (b) type B is 2.5 m long, 2 m wide, and 2 m high. The floors of both were bedded by a 15 cm thick layer of dry rice bran that was refreshed every 25 days; Figure S2. Schematic of the video recording process; Figure S3. Data collection angle, the cameras are recorded from the calf cage of the (a) top view in the middle; (b) top front; (c) front left; (d) front right; Figure S4. Calf Farming Breeding Environment.
