# Supplementary material for: Analysis and Comparison of New-Born Calf Standing and Lying Time Based on Deep Learning
Source: Animals (Basel). 2024 Apr 29;14(9):1324. doi: 10.3390/ani14091324 (PMC11083583; doi:10.3390/ani14091324)
Supplement: Supplementary file 1 [file animals-14-01324-s001.zip › animals-2985220-supplementary.pdf]

Experimental setup instructions

In this experiment, two types of calf cage A and B were used, as shown in Figure S1.

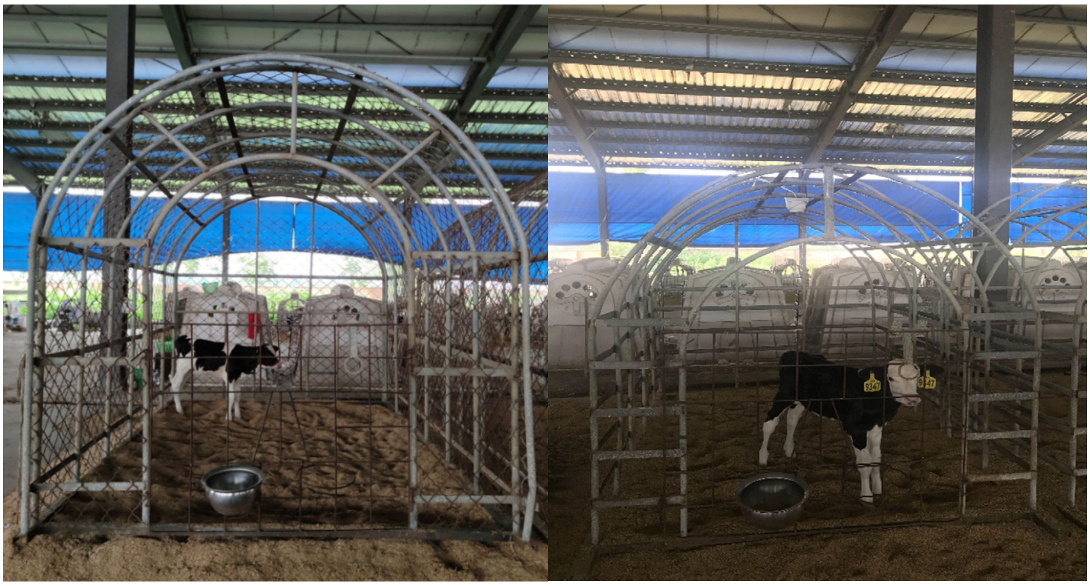

(a) (b)

Figure S1. Two types of cages used on the farm. (a) type A is 3 m long, 2.5 m wide, and 3 m high. (b) type B is 2.5 m long, 2 m wide, and 2 m high. The floors of both were bedded by a 15 cm thick layer of dry rice bran that was refreshed every 25 days.

The data collection experimental process in this study is shown in Figure S2, In the experiment, six cameras were mounted at different locations on the six calf cages (only one camera at different locations in the figure was installed in each calf cage in the experiment) and were used to record the daily behavioral changes of the six calves from different angles. For the two different calf cages, type A and type B, cameras were mounted at four positions of the calf cage. The installation of the camera is shown in Figure S2 at four positions (Top, Up, Side, and Side). For the installation of cameras at the side position, A-cage cameras were mounted on the left and right sides of the calf cage at a height of 2 m above the ground, and B-

cage cameras were mounted on the left and right sides of the calf cage at a height of 1.6 m above the ground. A camera (Top view camera) was mounted in the top center of the calf cage, allowing for overhead photography of calf behavior throughout the calf cage. The camera (Up-top view camera) was mounted directly above the front door of the calf cage, facing the calf in the cage. The installation of cameras in these four positions is based on the convenience of camera installation in daily breeding. This allowed the camera to capture the entire field of view (FOV) of the calves' activities in the calf cage. The installation of the camera ensured that the position of the camera did not adversely affect the behavior and life of the calves. In summary, for the recording unit of this experiment, there were three types of camera positions ('T', 'U', 'S') defined as follows：

T: Top view camera，overhead camera directly above; camera front down.

U: Up-top view camera; the camera is located above the front doors, with a 60-degree overhead field of view facing the calving cage.

S: Side view camera，the camera installed in the middle and upper position of the side pillar of the calf cage, the camera field of view facing the calf cage.

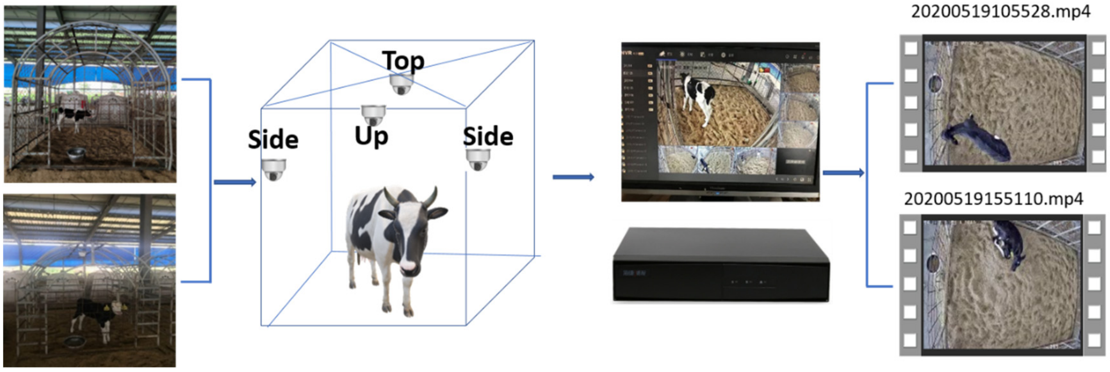

Figure S2. Schematic of the video recording process

The actual data collection scenario is shown in Figure S3

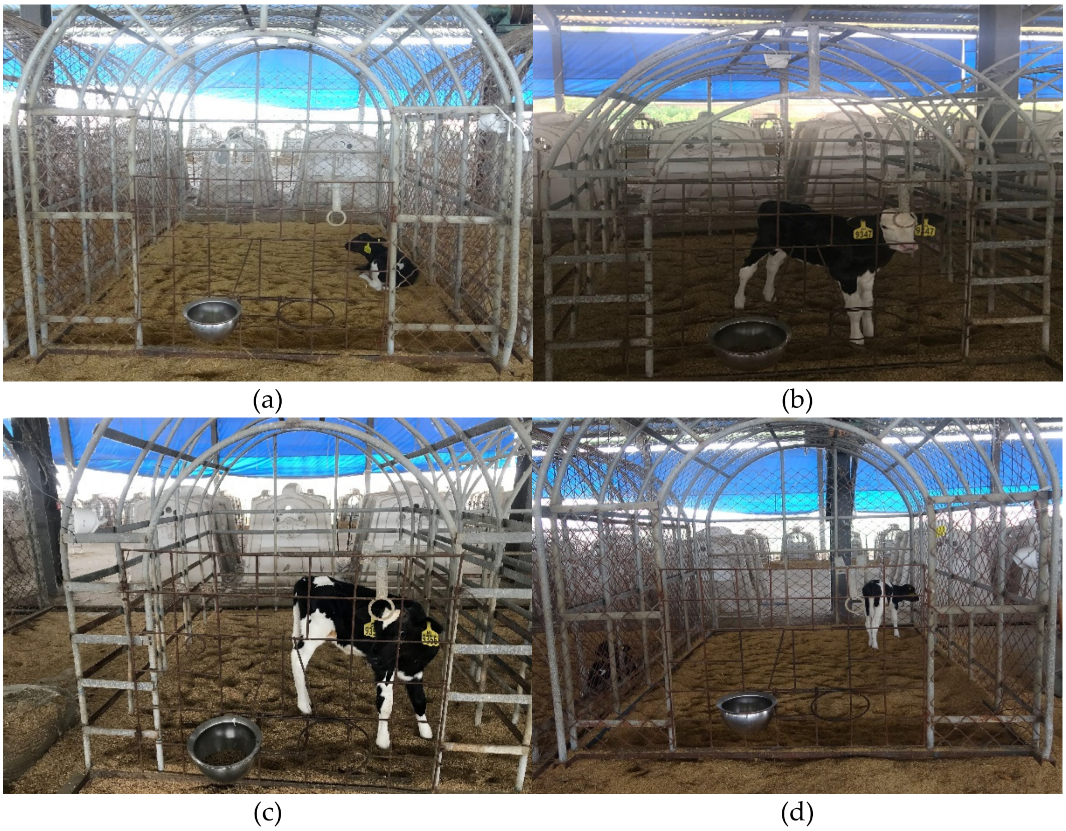

Figure S3. Data collection angle, the cameras are recorded from the calf cage of the (a) top view in the middle; (b) top front; (c) front left; (d) front right.

The real environment for breeding calves on the farm is shown in Figure S4.

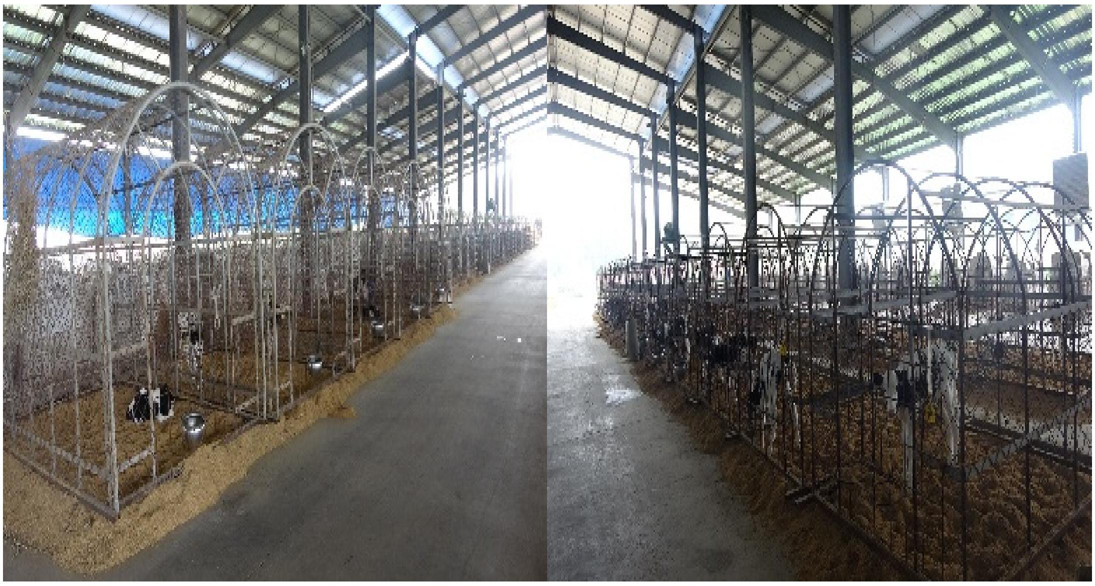

Figure S4. Calf Farming Breeding Environment
